# MC-H-Geo: A Multi-Scale Contextual Hierarchical Framework for Fine-Grained Lithology Classification

**DOI:** 10.3390/s25226859

**Published:** 2025-11-10

**Authors:** Lang Liu, Yanlin Shao, Yaxiong Shao, Peijin Li, Qingqing Yang, Rui Zeng

**Affiliations:** 1 School of Geosciences, Yangtze University, Wuhan 430100, China; 2022710470@yangtzeu.edu.cn (L.L.); 2023710527@yangtzeu.edu.cn (P.L.); 2024720560@yangtzeu.edu.cn (Q.Y.); 2023710537@yangtzeu.edu.cn (R.Z.); 2 Hubei Provincial Engineering Research Center for Unconventional Oil & Gas Geology and Engineering, Wuhan 430100, China; 3 Center for Governmental Studies, Northern Illinois University, DeKalb, IL 60115, USA; yshao1@niu.edu

**Keywords:** lithology classification, TLS point clouds, multi-scale contextual learning, hierarchical model, geological mapping

## Abstract

**Highlights:**

This study develops MC-H-Geo, a multi-scale contextual hierarchical framework for fine-grained lithological classification of TLS outcrop point clouds. The framework integrates voxel-based anchor point construction, a multi-scale feature engine with cross-scale differentials, a gated expert classifier with task-adaptive feature subsets, and a two-step geological post-processing strategy. The method is validated on the Qianwangjiahe outcrop dataset, achieving state-of-the-art performance and highlighting the physical limits and pointing to multi-sensor solutions.

**What are the main findings?**

- MC-H-Geo significantly improves fine-grained classification accuracy (OA = 94.3%, Macro F1 = 0.944), outperforming PointNet++, SG-RFGeo, and XGBoost.
- Error analysis demonstrates that misclassification of weathered sandstone as vegetation is caused by feature aliasing, marking a fundamental limitation of TLS-derived attributes.

**What is the implication of the main finding?**

- The framework offers a transferable solution for high-resolution digital outcrop modeling in fine-grained successions.
- Overcoming TLS-only feature aliasing requires multi-sensor data fusion (RGB, hyperspectral) and advanced deep learning architectures, pointing to the next frontier in automated geological mapping.

**Abstract:**

High-resolution lithological mapping of outcrops is fundamental for reservoir characterization and petroleum geology, yet distinguishing lithologies with subtle petrophysical contrasts remains a major challenge. This study proposes MC-H-Geo, a multi-scale contextual hierarchical framework for automated lithology classification from terrestrial laser scanning (TLS) point clouds. The framework integrates three modules: (i) a multi-scale contextual feature engine that extracts spectral, geometric, and textural descriptors across local and stratigraphic contexts, enhanced by cross-scale differentials to capture stratigraphic variability; (ii) a gated expert classifier with task-adaptive feature subsets for hierarchical vegetation–rock and intra-rock discrimination; and (iii) a two-step geological post-processing procedure that enforces stratigraphic continuity through *Z*-axis correction and neighborhood smoothing. Experiments on the Qianwangjiahe outcrop (Ordos Basin, China) demonstrate state-of-the-art performance (OA = 94.3%, Macro F1 = 0.944), outperforming PointNet++ (77.1%), SG-RFGeo (74.2%), and XGBoost (61.7%). Error analysis reveals that residual sandstone–vegetation confusion results from feature aliasing in weathered zones, highlighting the intrinsic limitations of TLS-only data. Overall, MC-H-Geo establishes an advanced framework for fine-grained lithological mapping and identifies multi-sensor data fusion as a promising pathway toward robust, geologically consistent outcrop interpretation.

## 1. Introduction

Outcrop analogs play a fundamental role in petroleum geology and reservoir characterization, serving as natural laboratories that link limited well data with three-dimensional subsurface models. Detailed lithological and sedimentological analyses of outcrops provide critical constraints for reconstructing depositional processes, predicting reservoir heterogeneity, and improving hydrocarbon recovery efficiency [1]. This demand is particularly pronounced in continental tight reservoirs, where the spatial distribution of fine-scale depositional microfacies exerts a strong influence on diagenesis, pore structure, and overall reservoir quality [2,3,4]. Therefore, achieving high-resolution and spatially continuous lithological identification at the microfacies scale represents both a fundamental scientific goal and a practical necessity for reducing uncertainties in exploration and development.

The emergence of terrestrial laser scanning (TLS) has revolutionized outcrop studies by delivering millimeter-resolution point clouds that capture both geometric and spectral (laser intensity) attributes [5,6]. These datasets form a powerful basis for machine learning (ML)-based lithological classification. However, while discriminating lithologies with strong petrophysical contrasts (e.g., limestones vs. shales) is now feasible, distinguishing subtle lithological transitions among spectrally similar rock types, such as the sandstones, siltstones, and mudstones common in clastic sedimentary systems, remains a formidable challenge [7,8].

This challenge is compounded by a suite of post-depositional geological processes and surface degradation mechanisms that alter or obscure the rock’s intrinsic spectral and geometric properties [9]. For instance, fine-grained sandstones and siltstones in outcrop settings are susceptible to physical weathering, which leads to granular disaggregation and increased surface roughness [10]. Furthermore, biological colonization by lichens and mosses introduces another layer of noise that can mimic the spectral signatures of certain rock types. Collectively, these degradation phenomena often cause the features of distinct lithologies to converge in the feature space—a problem known as feature aliasing. Moreover, geological structures such as minor faults, fractures, and complex bedding geometries further complicate automated interpretation by disrupting stratigraphic continuity [11]. This limitation reduces the ability of existing workflows to resolve heterogeneity at the resolution required for reservoir modeling.

Current TLS-based approaches generally fall into two directions. Spectral-based methods infer mineralogical composition from laser intensity, which works well when reflectance differences are distinct [12,13]. Geometric descriptors such as roughness and curvature capture surface morphology [14,15,16]. Fusing spectral and geometric features improves classification compared to single-feature strategies [17], while deep learning methods such as PointNet attempt to learn directly from raw point clouds [18,19]. Yet, TLS data are often degraded by vegetation cover, weathering, and acquisition geometry. Deep models tend to be sensitive to such noise and often emphasize global over local details, limiting robustness in geologically complex settings [20,21].

A critical drawback shared by most existing workflows is reliance on fixed-scale feature extraction. Typically, descriptors are computed within uniform neighborhoods (e.g., 10 cm spheres) [22,23]. While effective for local texture, this strategy neglects the inherently hierarchical nature of sedimentary systems, where diagnostic features span from millimeter-scale grains to meter-scale bedding [24]. As a result, fixed-scale descriptors fail to represent gradual or cross-scale transitions, forming a major bottleneck for automated fine-grained mapping [25]. Post-processing methods, such as smoothing filters or majority voting, can mitigate noise but risk erasing meaningful boundaries [26,27]. Conversely, corrections based on manually interpreted stratigraphic surfaces impose strong constraints but sacrifice automation and adaptability in environments with rapid lateral facies changes [6]. Moreover, any post-processing approach is inherently limited by the quality of the initial classification: systematic errors in the preliminary stage are difficult to eliminate downstream.

To overcome these interconnected challenges of multi-scale representation, robust classification, and geologically plausible refinement, we introduce a comprehensive framework for fine-grained lithological identification, termed MC-H-Geo (Multi-scale Contextual Hierarchical Geological framework). The framework is designed to maximize performance at each stage through three key innovations:

1. Multi-scale Contextual Feature Engine: Features are extracted at both fine (10 cm) and broader (30 cm) neighborhood sizes, supplemented by differential and ratio-based descriptors that explicitly quantify cross-scale variations. This design provides richer contextual information to capture the subtle signatures of microfacies-scale heterogeneity.
2. Task-Adaptive Hierarchical Modeling: A two-stage structure decomposes the classification workflow into specialized tasks—first vegetation–rock separation, then fine-grained lithological classification (sandstone, siltstone, mudstone). Recursive feature elimination (RFECV) is employed to optimize a dedicated feature subset for each stage, enhancing both discriminative power and model robustness.
3. Two-Step Geological Refinement and Boundary Analysis: A novel post-processing module first reconstructs stratigraphic continuity through a *Z*-axis layer sweep, then sharpens local details via neighborhood smoothing. Beyond performance gains, this analysis reveals an important insight: in weathered zones, lithological signals may overlap or blur due to erosion-induced surface mixing, defining the intrinsic physical limitation of TLS-only classification. and underscoring the need for multi-sensor data fusion in future studies.

Field validation at the Qianwangjiahe deltaic outcrop confirms that MC-H-Geo substantially improves the accuracy and continuity of lithological mapping at the microfacies scale. More broadly, this study makes three contributions: (i) Conceptual novelty: establishing a multi-scale contextual feature engine for TLS-based classification. (ii) Methodological advance: developing a task-adaptive hierarchical workflow tailored to the complexities of fine-grained discrimination. (iii) Scientific insight: defining the performance boundary of TLS-only lithological mapping, thereby motivating the integration of complementary remote sensing modalities.

Through these innovations, MC-H-Geo advances TLS-based lithological studies by providing a framework that is multi-scale, task-adaptive, and geologically consistent. The results enhance classification accuracy, improve stratigraphic interpretability, and highlight future directions for multi-source remote sensing of outcrops and reservoirs.

## 2. Study Area and Data

### 2.1. Study Area

**Figure 1:**
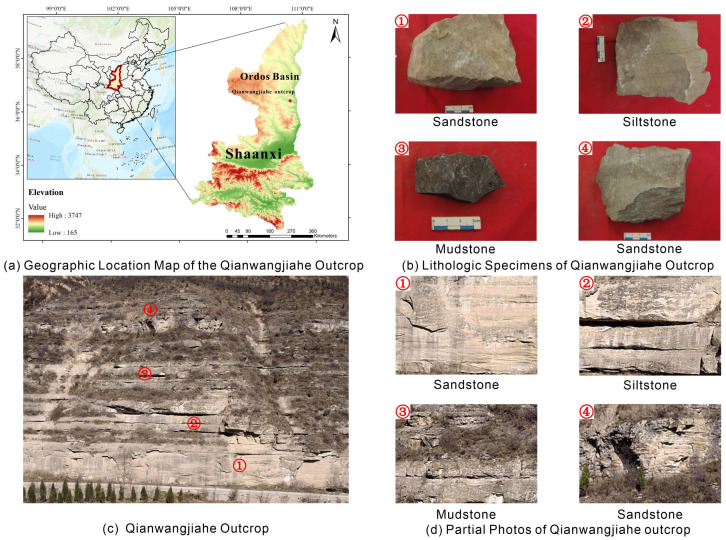
Regional and geological context of the Qianwangjiahe outcrop in the eastern of the Ordos Basin. (**a**) Geographic location map of the Qianwangjiahe outcrop within the Ordos Basin. (**b**) Lithologic specimens of Qianwangjiahe outcrop: ① sandstones of the delta plain (DP), ② siltstones of subaerial levee (LV), ③ mudstones of the delta front (DF), ④ sandstone of the subaqueous distributary channel (SCH). (**c**) Field photo of the Qianwangjiahe outcrop. (**d**) Partial high-resolution photos of different lithologies at the outcrop: ① sandstone, ② siltstone, ③ mudstone, ④sandstone.

The study area is the Qianwangjiahe outcrop, situated on the eastern margin of the Ordos Basin in Shaanxi Province, China (Figure 1a). The exposed strata belong to the Upper Triassic Yanchang Formation (T_3_y), a critical hydrocarbon-bearing unit in the basin. Geologically, the outcrop represents a series of meandering delta deposits, primarily composed of three lithologies: mudstone, sandstone, and siltstone. The lower part of the outcrop consists of a series of meandering delta deposits, including subaerial distributary channel (DCH) sandstones of the delta plain (DP) (Figure 1b①), subaerial levee (LV) siltstones (Figure 1b②), interdistributary bay (BA) mudstones of the delta front (DF) (Figure 1b③), and subaqueous distributary channel (SCH) sandstones (Figure 1b④).

Overall, the outcrop is well exposed, with distinct lithological assemblages and clearly recognizable stratigraphic contacts, providing favorable conditions for high-precision lithological identification. However, the depositional environment of this section is relatively stable, resulting in lithologies that are macroscopically similar. The strata are dominated by gray-green and yellow hues, while the distinction between sandstone and siltstone is primarily reflected in subtle grain-size variations. These subtle grain-size variations are below the effective resolution of TLS intensity data and are often masked by weathering crusts, making them nearly indistinguishable through conventional spectral or simple geometric analysis. This challenge necessitates the development of advanced feature extraction methods capable of capturing fine-scale textural and contextual information.

### 2.2. Data Acquisition and Preprocessing for Multi-Scale Analysis

#### 2.2.1. Data Acquisition

Terrestrial laser scanning (TLS) was conducted using a RIEGL VZ-400 system, which provides a ranging accuracy of ±5 mm and a pulse repetition rate up to 1200 kHz. Its key technical specifications are listed in Table 1. It is important to note that this instrument captures geometric coordinates and laser return intensity, but does not have an integrated camera to provide co-registered RGB (color) information for each point. Therefore, the primary data source for our automated classification is inherently monochrome. To ensure full coverage of the outcrop, three scanning stations were strategically arranged around the study area, with an average distance of 40–60 m from the cliff surface. The mean point density on the outcrop surface exceeded 1200 points/m^2^, enabling the capture of fine-scale structural and textural features.

**Figure 2:**
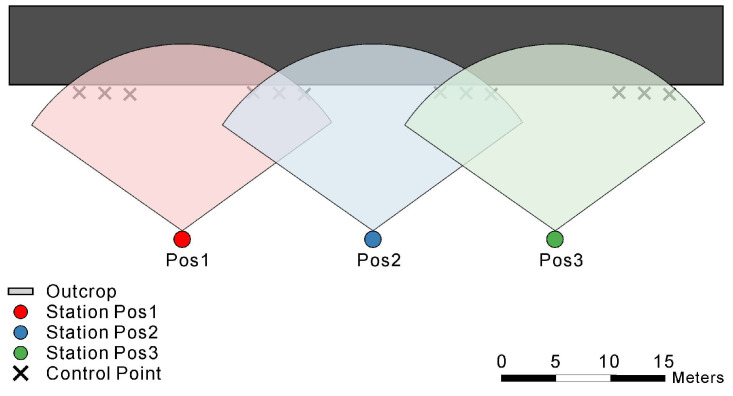
Terrestrial laser scanning (TLS) station distribution and scanning layout around the outcrop. Stations (Pos1–Pos3) were arranged to ensure complete coverage, with overlapping fields of view and a control point for registration.

To facilitate the manual labeling process and provide qualitative validation, high-resolution digital photographs were acquired separately from each scanning station using a DSLR camera. These photographs were used to generate a photorealistic 3D model that served as a visual reference for expert annotation. All scans were co-registered and georeferenced in RiSCAN PRO using both natural and artificial tie points. Noise reduction and outlier removal were performed, resulting in a high-quality dataset containing 95,564,072 valid point samples. The scanning geometry is shown in Figure 2.

#### 2.2.2. Anchor Point Construction

A key distinction of our methodology from conventional voxel-based approaches lies in the construction of the analytical units. To enable the proposed multi-scale contextual analysis, a set of analytical anchor points was generated.

**Figure 3:**
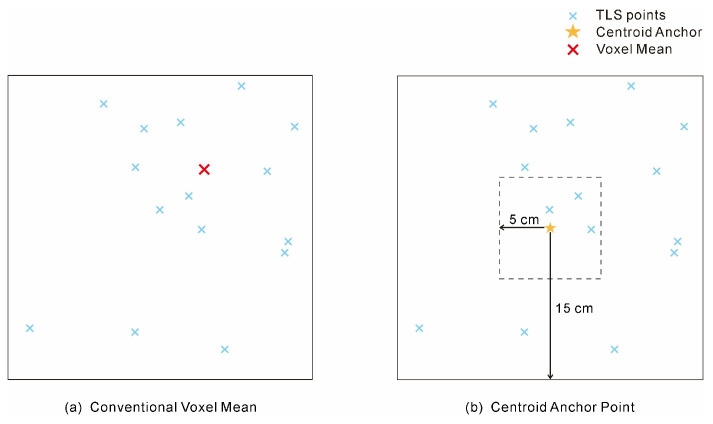
Comparison of two strategies for defining analytical units. (**a**) The conventional fixed-voxel method, where features are calculated from the point set within a pre-segmented 10 cm grid cell, represented by its centroid (Voxel Mean). (**b**) The proposed centroid-anchor-based method, where the voxel’s centroid is first determined to serve as an anchor point for the subsequent extraction of multi-scale neighborhoods from the raw point cloud.

Based on the observation that the thinnest siltstone layer at the Qianwangjiahe outcrop is approximately 1 cm, and considering both the instrument resolution and scanning step size, the entire point cloud was initially voxelized into cubic cells of 10 × 10 × 10 mm. This preliminary partitioning was not intended for direct feature extraction, but rather served as a systematic approach to generate a spatially representative set of sampling locations. For each non-empty voxel, the geometric centroid (x, y, z coordinates) of all contained points was calculated (Figure 3a). This process yielded a comprehensive set of anchor points, each representing a unique, data-driven position on the outcrop surface (Figure 3b). Compared with direct voxel-mean feature computation, this design provides two advantages: (1) it reduces the impact of uneven point density, resulting in a more uniform sampling grid; and (2) it produces representative anchor positions that more consistently preserve lithological boundaries. The generated anchor points form the basis for subsequent data labeling and multi-scale feature extraction.

#### 2.2.3. Data Labeling

The ground truth dataset for supervised learning was constructed by manually labeling a subset of the generated anchor points. The labeling process was guided by a combination of field observations and the interpretation of co-registered high-resolution digital imagery. To ensure high fidelity, the manual annotation was further calibrated and validated against the results from thin-section analysis of 54 representative rock samples collected from the outcrop. Each anchor point was ultimately assigned to one of four predefined classes: mudstone, sandstone, siltstone, or vegetation.

In total, 26,910 anchor points were labeled. As detailed in Table 2, the resulting class distribution is well-balanced, which is critical for mitigating model bias and ensuring the development of a robust and generalizable classification model.

The generated anchor points, together with their manually assigned lithological labels, serve as the input units for the subsequent analysis. Building upon these anchor points, the next step is to design a feature extraction strategy that captures lithological variability across multiple spatial scales, as detailed in Section 3.2.

## 3. Methods

### 3.1. Overall Framework

**Figure 4:**
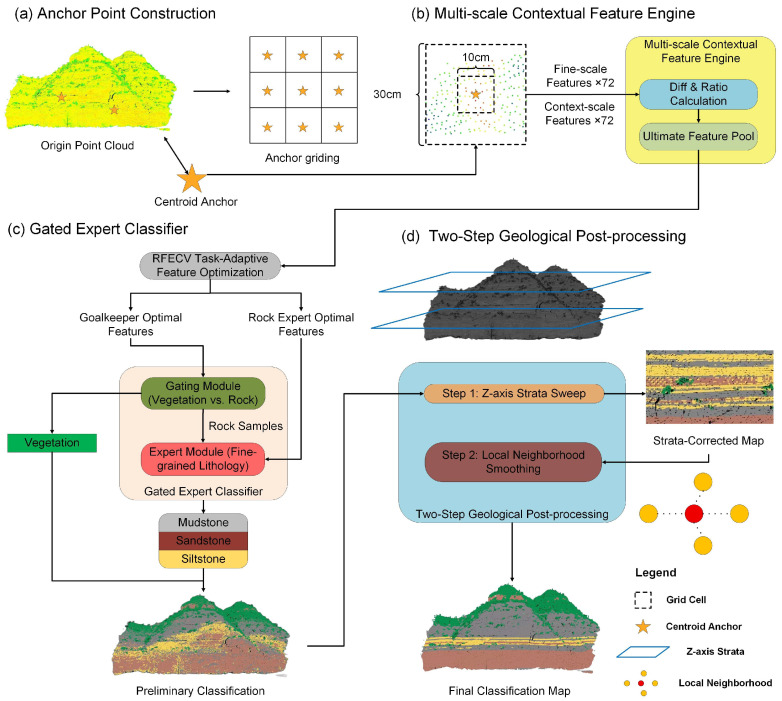
Overall workflow of the proposed MC-H-Geo framework: (**a**) anchor point construction (see Section 2.2), which generates representative analytical units from the raw outcrop point cloud; (**b**) multi-scale contextual feature engine, where spectral, geometric, textural, and frequency-domain descriptors are extracted at 10 cm and 30 cm neighborhoods and enhanced with scale-differential features; (**c**) gated expert classifier, which applies task-adaptive feature subsets for vegetation–rock separation and fine-grained lithology classification; and (**d**) two-step geological post-processing, consisting of *Z*-axis stratigraphic correction and local neighborhood smoothing to ensure geological plausibility.

The proposed MC-H-Geo framework is designed to enable fine-grained lithological classification from outcrop point clouds in complex geological settings. As illustrated in Figure 4, the workflow operates on the voxel-based anchor points established in Section 2.2 and integrates three successive methodological modules: a feature engineering engine, a hierarchical classifier, and a post-processing refinement module.

The Multi-scale Contextual Feature Engine first extracts a comprehensive set of spectral, geometric, textural, and frequency-domain descriptors at both fine (10 cm) and context (30 cm) scales, which are then enhanced with cross-scale differential features. Next, the Gated Expert Classifier employs a task-adaptive hierarchical strategy, utilizing optimized feature subsets to first separate vegetation from rock and then discriminate among the fine-grained lithologies. Finally, the Two-Step Geological Post-processing Module refines the preliminary classification map by applying *Z*-axis stratigraphic correction and local neighborhood smoothing to ensure geological plausibility. This design not only improves classification accuracy but also ensures that the results remain consistent with geological principles. The following sections provide a detailed description of each module.

### 3.2. Multi-Scale Contextual Feature Engine

Lithological heterogeneity in outcrop-scale point clouds is expressed across multiple spatial scales. Relying on a single neighborhood often leads to feature aliasing, where fine-scale variations are obscured by broader contextual patterns. To address this issue, the proposed framework incorporates a multi-scale contextual feature engine, which systematically extracts, enhances, and organizes features around voxel-based anchor points. The overall structure of the feature pool is summarized in Table 3.

Accordingly, the ultimate feature pool integrates five complementary categories of descriptors. Spectral statistics capture intensity variations of laser returns that are linked to mineral composition and surface reflectance properties. Geometric features describe local morphology and 3D structural anisotropy of the outcrop surface. Textural measures quantify neighborhood-level heterogeneity through second-order statistics, while frequency-domain descriptors reveal periodic patterns and energy distributions not evident in the spatial domain. Finally, multi-scale differential features explicitly encode cross-scale contrasts, enhancing sensitivity to lithological transitions at boundaries. By combining these categories into a unified, multi-dimensional representation, the feature engine provides a robust basis for subsequent task-adaptive classification.

#### 3.2.1. Multi-Scale Feature Extraction

**Figure 5:**
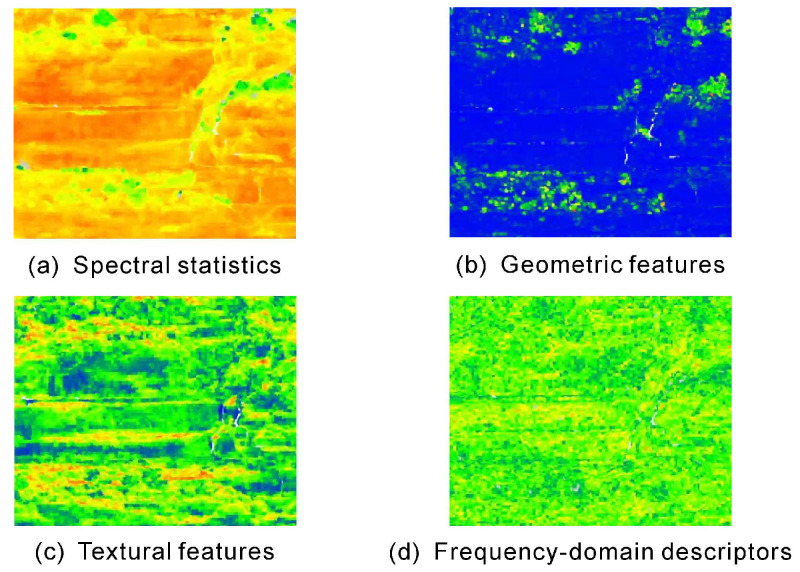
Representative visualizations of multi-scale features included in the ultimate feature pool. Examples are shown for four feature categories: (**a**) spectral statistics (mean reflectance at 30 cm scale), (**b**) geometric features (sphericity at 10 cm scale), (**c**) textural measures (GLCM contrast of reflectance at 30 cm scale), and (**d**) frequency-domain descriptors (FFT peak ratio of reflectance at 10 cm scale). The color scale (from blue/low to red/high values) highlights how these different descriptors capture distinct spatial patterns corresponding to underlying lithological variations. Together with the cross-scale differential features (see Section 3.2.2), they constitute the ultimate feature pool summarized in Table 3.

The engine’s foundational step is the extraction of base features from two concentric cubic neighborhoods defined around each anchor point: a fine-scale (10 cm) and a context-scale (30 cm). The two neighborhood scales (10 cm and 30 cm) were selected to match the characteristic lithological variability of the outcrop. The fine-scale window captures sublayer textures and thin interbeds (1–2 cm), while the context-scale window integrates broader structural trends (20–40 cm) without oversmoothing. Within each neighborhood, four categories of features were computed to capture complementary aspects of spectral, geometric, and structural variability. Representative examples of each category are visualized in Figure 5 to illustrate their distinct responses to lithological variability across mudstone, sandstone, siltstone, and vegetation classes.

Spectral statistics were derived from both reflectance and amplitude distributions, including mean, standard deviation, skewness, kurtosis, interquartile range, and number of peaks. To mitigate acquisition bias, both raw and normalized reflectance(refnorm) were used, the latter weighting each return by its squared distance to the neighborhood centroid to enhance peripheral responses. These descriptors reflect compositional and textural differences in surface materials—mudstones typically exhibit low, variable reflectance due to clay minerals and weathering coatings, whereas quartz-rich sandstones and siltstones yield higher and more stable responses [13,28].

Geometric features quantify local surface morphology and anisotropy through roughness measures (at multiple radii), eigenvalue-based shape indices (linearity, planarity, sphericity), and point density. These parameters encode depositional and diagenetic signals: mudstones display smooth, planar surfaces associated with fine-grained, low-energy deposition, while sandstones and siltstones exhibit higher roughness and greater anisotropy due to coarser grains and variable cementation [29,30].

Textural features, computed from gray-level co-occurrence matrices (GLCM) of reflectance and amplitude, characterize second-order spatial statistics including contrast, dissimilarity, homogeneity, energy, and correlation. Fine-grained mudstones yield high homogeneity and low contrast, whereas coarser sandstones and siltstones show stronger dissimilarity and energy due to their heterogeneous microstructure [31,32]. These descriptors effectively bridge spectral composition and geometric morphology by quantifying local heterogeneity.

Frequency-domain descriptors were obtained via fast Fourier transform (FFT) to capture periodicity and spectral energy distributions. Metrics such as spectral energy, entropy, peak ratio, and energy fractions across low-, mid-, and high-frequency bands highlight the internal organization of outcrop surfaces. Sandstones with bedding or lamination often exhibit dominant peaks in low–mid frequencies, while mudstones show higher entropy and more diffuse spectral responses, indicative of homogeneous fabric [33].

#### 3.2.2. Cross-Scale Differential Features

While the single-scale features described above effectively capture the intrinsic properties of anchor points, certain classification ambiguities persisted when relying solely on absolute descriptors. For instance, weathered mudstone surfaces near the rock–vegetation boundary often produced sparse returns with weak reflectance at the 10 cm scale, resembling true vegetation. Similarly, subtle textural contrasts between sandstone and siltstone were frequently masked at a single analytical scale.

To address these issues, we designed a set of cross-scale differential features that explicitly encode relative changes between fine (10 cm) and context (30 cm) neighborhoods. For each base feature *f*, two differential forms were computed:

(1) $$\Delta f=f_{30cm}-f_{10cm},\;R_{f}=\frac{f_{30cm}}{f_{10cm}+\varepsilon }$$

where $f_{10cm}\;$and $f_{30cm}$ denote values at the fine (10 cm) and context (30 cm) scales, respectively, and ε is a small constant to prevent division by zero. The difference term emphasizes absolute variability across scales, while the ratio captures relative contrasts. These features enhance sensitivity to boundary transitions and lithological heterogeneity that may be overlooked by single-scale descriptors.

**Figure 6:**
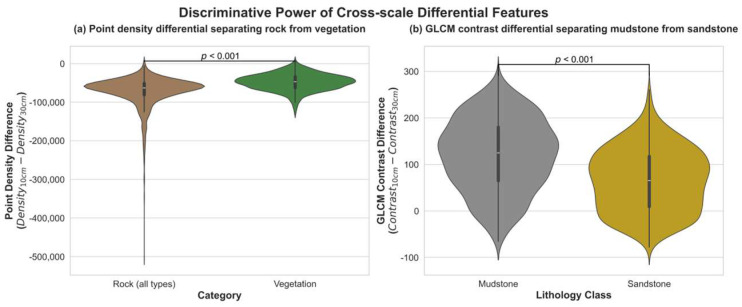
Discriminative power of cross-scale differential features in resolving classification ambiguities. (**a**) Point density difference distinguishing rock from vegetation. Ambiguous rock samples that were misclassified as vegetation in the single-scale feature space exhibit density differentials comparable to typical rocks, enabling their separation from true vegetation (*p* < 0.001). (**b**) GLCM reflectance contrast difference separating mudstone from sandstone. Sandstone samples show systematically higher differentials than mudstones, reflecting greater heterogeneity at broader scales (*p* < 0.001).

As illustrated in Figure 6, differential metrics substantially improve class separability. In panel (a), the point_density_diff feature effectively distinguishes vegetation from weathered rock: while vegetation maintains consistently low density across scales, weathered mudstone transitions from sparse fine-scale returns to denser distributions at the context scale, producing a strong positive differential. In panel (b), the cross-scale GLCM contrast differential (*glcm_ref_contrast_diff*) provides a statistically significant discriminator between mudstone and sandstone, reflecting the higher heterogeneity and coarser grain fabric of sandstone when analyzed at broader scales.

These findings demonstrate that cross-scale differentials encode meaningful geological variability by combining fine-scale precision with broader contextual cues. When integrated with the single-scale spectral, geometric, textural, and frequency-domain descriptors, they form a comprehensive Ultimate Feature Pool (Table 3), enabling the subsequent classifier to leverage both intrinsic attributes and scale-dependent contrasts for more robust lithological discrimination.

### 3.3. Gated Expert Classifier

**Figure 7:**
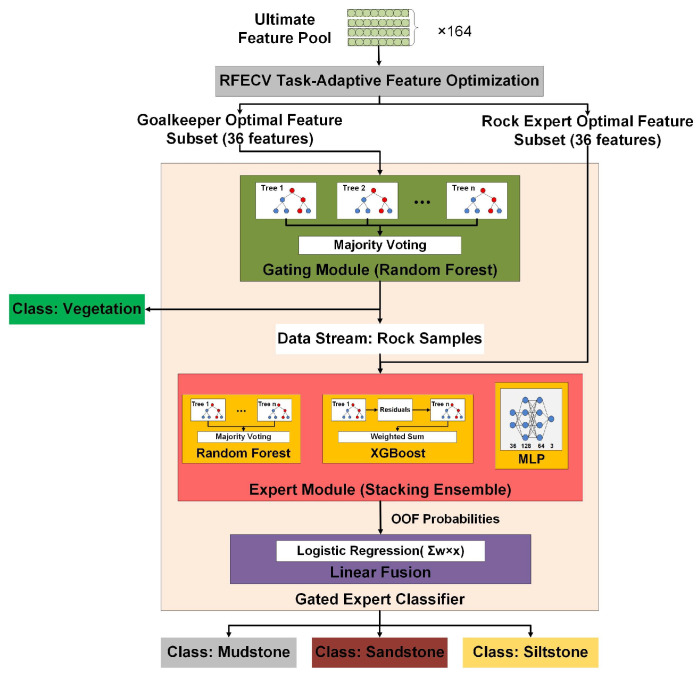
Structure of the Gated Expert Classifier. The two-stage architecture first employs a Random Forest–based Gating Module for vegetation–rock separation, followed by a stacking-based Expert Module that integrates Random Forest, XGBoost, and MLP branches, with a Logistic Regression meta-learner fusing their outputs for fine-grained lithological classification.

The high dimensionality of the constructed feature pool and the heterogeneous nature of lithological classification render a single-layer model inadequate for achieving stable and interpretable results. Specifically, the strong and distinct spectral-geometric signatures of vegetation, while easy to identify, can dominate the learning process. This risks obscuring the subtle, yet geologically critical, variations required to distinguish fine-grained clastic lithologies from one another. To address this, we developed a Gated Expert Classifier, a two-stage hierarchical framework that decomposes the complex multi-class problem into specialized subtasks (Figure 7), Such a hierarchical approach allows each module to operate on an optimized feature subset tailored to its specific challenge, thereby enhancing both discriminative power and geological interpretability.

#### 3.3.1. Task-Adaptive Feature Optimization

A single, unified feature set is inadequate for simultaneously handling vegetation–rock separation and fine-grained lithological discrimination, as these subtasks differ fundamentally in class separability and feature relevance. To address this issue, we implemented a task-adaptive feature optimization strategy based on Recursive Feature Elimination with Cross-Validation (RFECV). This ensures that each module within the Gated Expert Classifier operates on the most discriminative yet compact subset of features.

Formally, given the complete feature pool *F*, RFECV seeks the subset S^*^ that maximizes a task-specific cross-validated score:

(2) $$S^{*}=\underset{S\subseteq F}{argmax}CV\left(M,S\right)$$

where *M* denotes the base estimator (Random Forest), and *CV(M*,*S)* represents its stratified five-fold cross-validated performance. Accuracy was used for vegetation–rock separation and macro F1-score for intra-rock discrimination.

The optimization proceeds iteratively by ranking feature importance, removing the least informative variables, and reassessing model performance until the subset yielding the highest cross-validation score is obtained. Applied independently to the two modules, this process produced distinct 36-feature subsets: the Gating Module emphasized spectral, geometric, and differential descriptors capturing strong vegetation–rock contrasts, whereas the Expert Module prioritized textural and frequency-domain features sensitive to fine-grained lithological differences. By tailoring feature selection to each classification stage, the task-adaptive design reduces redundancy, mitigates dimensionality effects, and enhances both computational efficiency and geological interpretability, offering superior robustness and accuracy over conventional unified feature selection schemes.

#### 3.3.2. Model Implementation

Following a divide-and-conquer design, the Gating Module uses a Random Forest (RF) classifier optimized for vegetation–rock separation. RF’s robustness to noise and non-linear decision boundaries enables reliable performance where vegetation returns overlap with weathered rock surfaces. Operating on the optimized “goalkeeper” feature subset (Section 3.3.1), the module ensures stable and interpretable separation without requiring feature standardization.

The Expert Module addresses the finer discrimination among mudstone, sandstone, and siltstone using a stacking ensemble of RF, XGBoost, and a Multi-Layer Perceptron (MLP):

- RF captures geometric–structural variability and broad morphological differences.
- XGBoost models non-linear feature interactions within spectral and differential descriptors.
- MLP provides flexibility in high-dimensional feature learning, complementing tree-based methods.

Their probability outputs are fused through a Logistic Regression meta-learner, which learns the optimal weighting to produce final lithological labels. This ensemble leverages the complementary strengths of individual models, yielding balanced and geologically meaningful decision boundaries.

Model hyperparameters were optimized via grid search with five-fold cross-validation, tuning the number of trees and maximum depth (RF), learning rate and regularization (XGBoost), and hidden-layer sizes (MLP). Final configurations are summarized in Table 4.

Overall, the Gated Expert Classifier achieves superior interpretability and robustness by explicitly separating vegetation–rock discrimination from intra-rock classification and equipping each with optimized feature subsets and classifiers. This hierarchical, task-adaptive framework substantially improves accuracy and geological consistency, representing a methodological advancement beyond earlier double-layer RF approaches.

### 3.4. Two-Step Geological Post-Processing

**Figure 8:**
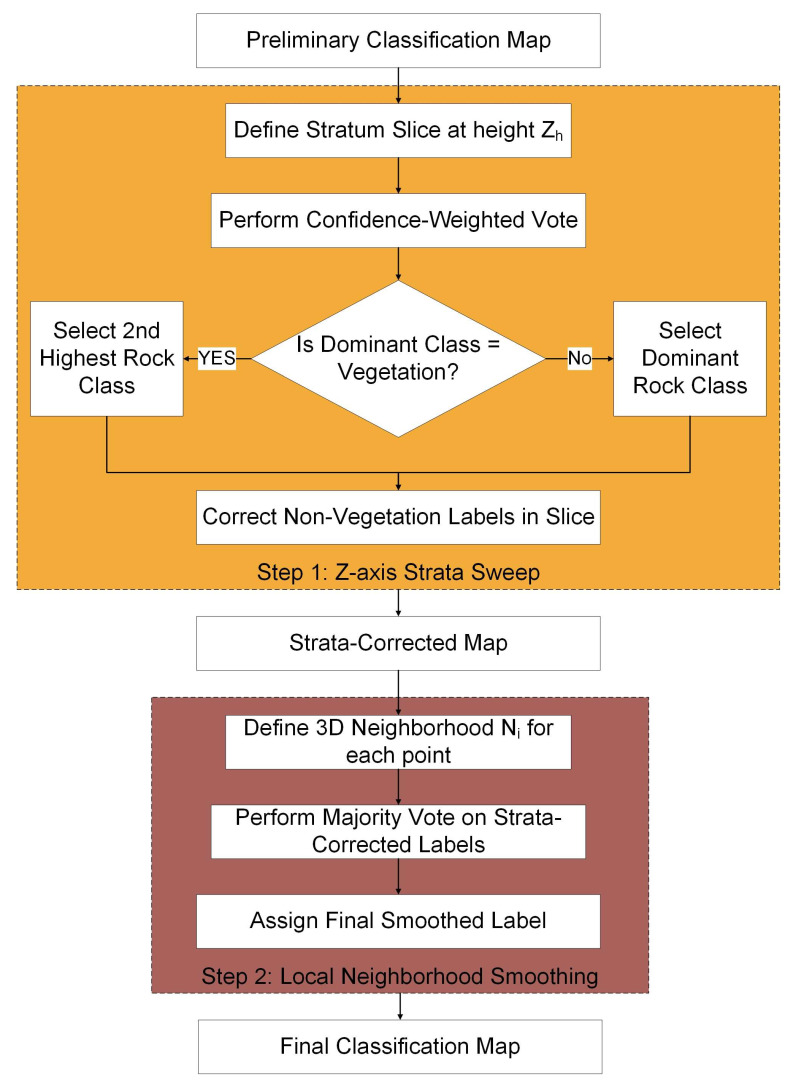
Workflow of the two-step geological post-processing.

Although the Gated Expert Classifier (Section 3.3) provides robust lithological predictions, the raw classification map may still exhibit local noise and mixed labels due to surface weathering, vegetation occlusion, and scanning artifacts. These inconsistencies, often manifested as “salt-and-pepper” noise, are most pronounced along lithological boundaries [33]. To address this issue, we developed a two-step geological post-processing strategy that explicitly integrates stratigraphic continuity and lithological uniformity (Figure 8). The synergy of this approach lies in its hierarchical design for error correction. The first step, a macroscopic correction via *Z*-axis Strata Sweep, is designed to enforce large-scale geological coherence. It primarily corrects isolated, geologically implausible misclassifications within otherwise continuous stratigraphic units by leveraging the principle of lateral continuity. The second step, Local Neighborhood Smoothing, then acts as a meso-scale refinement. It specifically targets the residual ‘salt-and-pepper’ noise, which is most prominent at the ambiguous boundaries between different lithologies, thereby sharpening the final contacts and ensuring local uniformity. By combining these two steps, the framework first corrects the overall stratigraphic architecture. It then refines the detailed geometric contacts, resulting in a more robust and geologically realistic classification map.

The workflow of the two-step geological post-processing is illustrated in Figure 8, where the two complementary steps are outlined as follows. Step 1: Macroscopic Correction via *Z*-axis Strata Sweep. This stage enforces vertical stratigraphic consistency by scanning the classified point cloud along the *Z*-axis, which corresponds to the primary depositional direction in the sub-horizontal strata of the study area. This is done using a vertical slicing interval of *Δz* = 0.1 m. This parameter was carefully chosen based on geological field observations. The thinnest siltstone interbeds in the Qianwangjiahe outcrop are approximately 10–15 cm thick. Setting Δz to 0.1 m ensures that these critical, thin stratigraphic units are resolved and not averaged out during the correction process. A significantly larger Δz (e.g., 0.5 m) would risk homogenizing distinct thin layers, leading to a loss of geological detail. Conversely, a significantly smaller Δz (e.g., 0.01 m) would make the process overly sensitive to point-level noise and might fail to capture the representative lithology of a slice. Therefore, Δz = 0.1 m represents a balanced choice that honors the geological scale of the target strata while maintaining robustness against noise. For each slice $S_{h}$, lithology-specific confidence values are accumulated and the dominant class is assigned according to:

(3) $$C_{i}^{\left(h\right)}=\sum_{p\in S_{h},pred\left(p\right)=i}confidence\left(p\right),\;L_{h}^{*}=\arg\underset{i}{max}C_{i}^{\left(h\right)}$$

If vegetation emerges as the dominant class within a slice, the second-ranked lithology is selected. This rule is based on the geological prior that vegetation is a surficial feature and cannot form a primary stratigraphic unit. While this heuristic is effective in our context for correcting misclassifications while preserving strata continuity, we acknowledge that it is a strong assumption. A more sophisticated implementation, as suggested, could incorporate an ‘unknown’ or ‘unclassified’ category. For instance, if the confidence gap between the vegetation class and the second-ranked lithology is very large, the slice could be flagged as ‘ambiguous’ rather than forcing a classification. This represents a promising avenue for future research to further improve the model’s robustness and honesty in highly complex or vegetated areas.

Step 2: Local Neighborhood Smoothing. To refine mesoscale geometry and remove voxel-level noise, each voxel $p_{u}$, is smoothed using majority voting within a spherical neighborhood of radius *R* = 0.5  m:

(4) $$N_{u}=\left\{\left.p_{v}\in p\left|\left\|p_{v}-p_{u}\right\|\right.\leq R\right\}\right.$$

The final smoothed label ${\hat{L}}_{u}$ is determined via majority voting over the strata-corrected labels in $N_{u}$:

(5) $${\hat{L}}_{u}=\arg\underset{i}{max}\sum_{p_{v}\in N_{u}}1\left(label\left(p_{v}\right)=i\right)$$

This step suppresses unrealistic cross-stratal mixing and reinforces lateral continuity in thinly interbedded mudstone–siltstone successions.

By combining *Z*-axis sweeping for macroscopic correction and neighborhood smoothing for mesoscopic refinement, the two-step post-processing integrates geological prior knowledge directly into the classification pipeline. It is important to note that while the current implementation using a fixed *Z*-axis is highly effective for the geological setting of this study, its generalization to areas with tilted or folded strata would require dip-aware adaptation, such as aligning the sweep direction with a locally computed bedding orientation. Similarly, our rule for replacing dominant vegetation classifications is based on the geological prior that vegetation is a surficial feature, not a stratigraphic unit. A more advanced implementation could introduce an ‘unknown’ or ‘unclassified’ category for such cases, especially where confidence scores are low, which remains a promising direction for future work.

## 4. Results and Analysis

### 4.1. Evaluation Metrics

To provide substantial and robust evidence for the performance of the proposed MC-H-Geo framework, we implemented a comprehensive validation strategy. Firstly, the evaluation is based on a large, high-fidelity ground truth dataset, comprising 26,910 manually labeled anchor points, with annotations guided by field observations and verified against thin-section analysis (as detailed in Section 2.2.3). Secondly, to assess model performance, we employed two distinct cross-validation schemes. For internal comparisons, such as the ablation study (Section 4.3), a standard stratified 5-fold random cross-validation was used. However, for the final comparative analysis against baseline methods (Section 4.4), we additionally implemented a more rigorous spatial block cross-validation to provide a conservative and realistic estimate of the model’s true generalization performance.

To quantitatively evaluate the performance of the proposed MC-H-Geo framework, a series of standard classification metrics derived from the confusion matrix were employed. The confusion matrix summarizes prediction outcomes across k classes, enabling the computation of both global and class-specific indicators.

Overall Accuracy (OA) measures the proportion of correctly classified voxels in the entire test set:

(6) $$OA=\frac{\sum_{i=1}^{k}TP_{i}}{N}$$

where TPi is the number of correctly classified samples of class i, k is the total number of classes, and N is the total number of test samples.

Class-wise performance is assessed using Precision and Recall:

(7) $$Precision_{i}=\frac{TP_{i}}{TP_{i}+FP_{i}},\;Recall_{i}=\frac{TP_{i}}{TP_{i}+FN_{i}}$$

where FP_i_ and FN_i_ denote false positives and false negatives for class i, respectively. Precision emphasizes commission errors (mislabeling), while recall highlights omission errors (missed detections).

The F1-score, defined as the harmonic mean of precision and recall, balances these two aspects:

(8) $$F1_{i}=\frac{2\cdot Precision_{i}\cdot Recall_{i}}{Precision_{i}+Recall_{i}}$$

To account for class imbalance—common in geological datasets where sandstone samples often dominate—the unweighted Macro-F1 is reported as the mean of all class-specific F1-scores. This ensures equal contribution from both major and minor lithologies.

In this study, model performance was evaluated using five-fold cross-validation to reduce randomness introduced by a single split. In each round, the dataset was stratified into 80% training and 20% validation subsets, preserving the class distribution. The average metric across folds was reported to minimize random variation and provide a robust estimate of generalization performance.

### 4.2. Feature Analysis and Selection Results

To optimize classification performance and understand the discriminative capacity of the engineered features, we conducted a systematic feature analysis and selection process. This step reduced dimensionality and identified key descriptors most effective for the hierarchical tasks of vegetation–rock separation and intra-rock discrimination.

**Figure 9:**
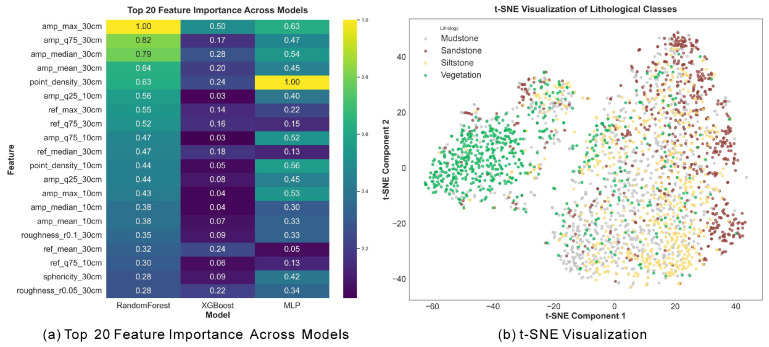
Feature importance and class separability.

As shown in Figure 9, the t-SNE projection indicates that vegetation forms a distinct cluster due to its unique spectral–geometric characteristics, while mudstone, sandstone, and siltstone exhibit significant overlap, confirming the challenge of fine-grained lithological discrimination. Feature importance analysis reveals that amplitude-related spectral features at the 30 cm scale (e.g., amp_max_30 cm, amp_q75_30 cm) dominate across models, followed by geometric indicators such as point_density_30 cm and sphericity_30 cm. These results highlight the combined contribution of spectral intensity and coarse-scale geometry to lithological separability.

**Figure 10:**
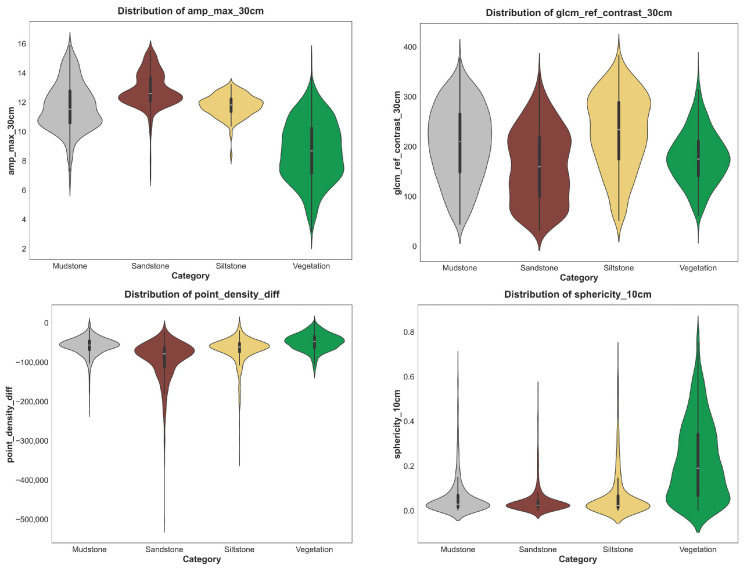
Representative feature distributions.

Figure 10 presents representative feature distributions. Spectral amplitude maxima effectively distinguish vegetation from rock, whereas geometric (sphericity_10 cm) and textural (glcm_ref_contrast_30 cm) features enhance differentiation among sandstone, mudstone, and siltstone. Cross-scale descriptors such as point_density_diff further improve boundary recognition by capturing relative variations between fine and context scales.

**Figure 11:**
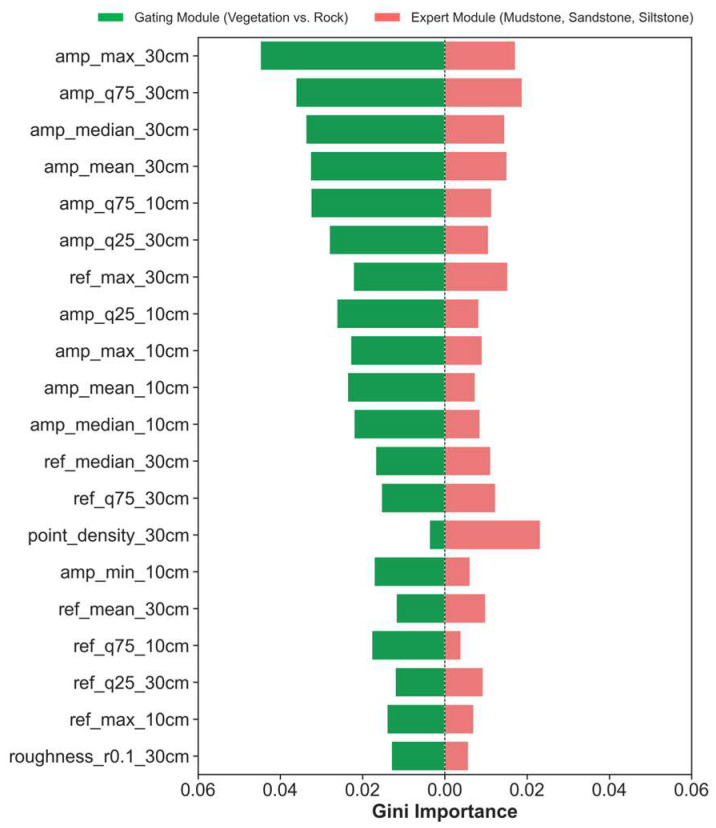
Comparative feature importance for hierarchical modules. The figure contrasts the dominant features used in the Gating Module (vegetation vs. rock) and the Expert Module (mudstone, sandstone, siltstone). Higher Gini importance values denote stronger contributions, emphasizing the task-specific feature requirements that justify the hierarchical classification design.

A module-wise comparison (Figure 11) demonstrates striking task-specific feature dependencies that are directly linked to the underlying physical properties of the targets. The Gating Module, tasked with separating vegetation from rock, predominantly relies on amplitude-based features at the 30 cm context scale (e.g., amp_max_30 cm, amp_q75_30 cm). This is geologically intuitive: vegetation, with its high moisture content and complex cellular structures, absorbs and scatters the near-infrared laser signal differently than dry, crystalline rock surfaces. Consequently, vegetation exhibits lower and more variable amplitude returns, making laser amplitude a highly effective discriminator. The importance of the larger (30 cm) scale suggests that the overall form and density of vegetation patches, rather than individual leaves, provide the most robust separation signal. Conversely, the Expert Module, which must resolve subtle intra-rock variations, leverages a more diverse suite of features. As Figure 11 shows, while some spectral features (ref_max_30 cm) remain important, geometric and textural descriptors gain significant prominence. For instance, point_density_30 cm becomes a key feature. This is because different lithologies, despite similar mineralogy, exhibit different weathering patterns and porosities, affecting point cloud density. Denser, less weathered sandstones will retain a higher point density than friable, easily weathered mudstones. Similarly, other geometric and textural features (not all shown in the top 20) capture critical differences in rock fabric, such as grain size, sorting, and bedding, which are the primary criteria for lithological discrimination in the field. After applying Recursive Feature Elimination with Cross-Validation (RFECV), both modules retained 36 optimized features, each emphasizing these distinct geological signals. For full reproducibility, the complete lists of these optimized feature subsets for both the Gating and Expert modules are provided in Appendix B (Table A3 and Table A4).

Finally, to ensure robust performance and avoid redundancy, Recursive Feature Elimination with Cross-Validation (RFECV) was applied to derive the optimal feature subsets for each module. This process reduced the ultimate feature pool to 36 features for both the Gating and Expert Modules, with notable differences in composition: the Gating Module subset emphasized amplitude-based descriptors, whereas the Expert Module subset incorporated a broader mix of spectral, geometric, and textural features. This task-specific feature tailoring demonstrates that the MC-H-Geo framework leverages geological knowledge and data-driven selection synergistically, ensuring that each classification stage is optimized for its respective discrimination challenge.

### 4.3. Ablation Study

To assess the effectiveness of the proposed MC-H-Geo framework, we conducted ablation experiments by progressively removing or replacing key modules under identical baseline data and evaluation metrics. Four experimental schemes were tested:

- Scheme I: Single-scale features (10 cm) with a single-layer Random Forest model.
- Scheme II: Multi-scale features (10 cm + 30 cm) including differential and ratio descriptors, combined with a single-layer Random Forest model.
- Scheme III: Multi-scale features with the Gated Expert Classifier (hierarchical structure and RFECV-based optimal feature subsets).
- Scheme IV: Full MC-H-Geo framework, combining multi-scale features, Gated Expert Classifier, and the Two-Step Geological Post-processing.

**Figure 12:**
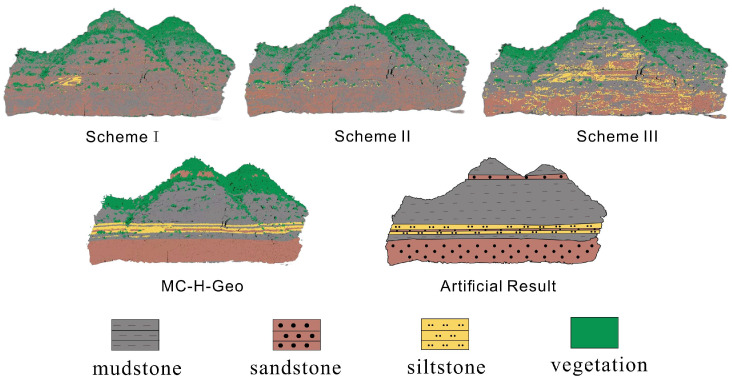
Ablation experiment results.

Figure 12 compares the spatial classification results of the four schemes against the expert geological interpretation. As model complexity increases, lithological boundaries become progressively clearer and stratigraphic continuity improves. Schemes I and II capture coarse vegetation–rock contrasts but show confusion between fine-grained lithologies, whereas Scheme III markedly improves intra-rock discrimination through hierarchical feature optimization. Scheme IV achieves the closest match to geological interpretation, producing continuous and geologically consistent strata with minimal noise.

Quantitative results (Table 5) show a steady performance improvement across schemes: overall accuracy (OA) increases from 0.692 (Scheme I) to 0.943 (Scheme IV), and Macro F1 rises from 0.699 to 0.945. This confirms that multi-scale features, hierarchical learning, and geological post-processing each contribute incrementally to accuracy and stability.

**Figure 13:**
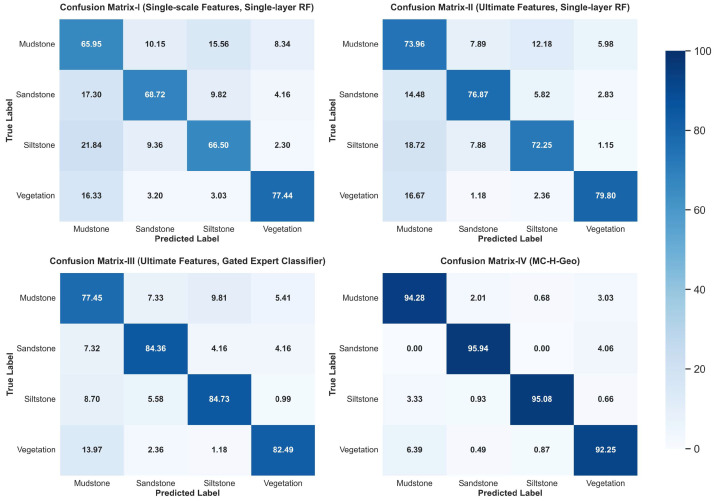
Ablation experiment confusion matrix.

Figure 13 presents class-level recall matrices. Scheme I struggles with sandstone–mudstone confusion (recall < 70%), while Scheme II slightly improves siltstone identification. Scheme III significantly boosts recall for all rock classes (>80%), and Scheme IV achieves balanced recall above 92% across all lithologies, demonstrating the critical role of geological post-processing in enforcing stratigraphic continuity and reducing boundary noise.

Overall, the ablation study demonstrates that (i) multi-scale features enhance fine-grained discrimination, (ii) the Gated Expert Classifier improves hierarchical robustness, and (iii) geological post-processing ensures geologically coherent lithological mapping.

### 4.4. Comparative Analysis and Performance Evaluation

The effectiveness of the proposed MC-H-Geo framework for fine-grained lithological classification was evaluated on the Qianwangjiahe outcrop dataset in the Ordos Basin against three baseline methods: XGBoost, PointNet++, and SG-RFGeo. XGBoost, a classical ensemble learning algorithm, is robust for high-dimensional feature tasks. PointNet++, a deep neural network for 3D point cloud segmentation, captures local geometric structures via hierarchical feature aggregation [17]. SG-RFGeo partitions point clouds into fixed-size voxels and classifies lithology based on statistical and geometric descriptors [8], but its reliance on uniform units limits adaptability in complex stratigraphy. Comparison with MC-H-Geo highlights the advantages of multi-scale features, hierarchical classification, and geological post-processing in improving accuracy and geological consistency.

Model parameters were tuned to ensure fairness. All baseline models were optimized using grid search with five-fold cross-validation conducted exclusively on the training set to avoid data leakage during hyperparameter tuning. For XGBoost, this resulted in 300 trees (maximum depth of 6), a learning rate of 0.05, and subsample/column sampling ratios of 0.9. PointNet++ processed 1024 points per sample, trained for 50 epochs with a batch size of 16 using the Adam optimizer (learning rate 0.001). SG-RFGeo employed a two-layer Random Forest: a coarse-level classifier with 100 trees (max depth 20) and a fine-level classifier with 200 trees (max depth 30), both with a minimum leaf size of 15. To ensure an unbiased comparison, all models were subsequently trained and evaluated on the same dataset under two independent validation schemes—random 5-fold cross-validation and spatial block cross-validation—using identical performance metrics. For both feature-based methods (XGBoost and SG-RFGeo), the complete Ultimate Feature Pool containing all 164 multi-scale and differential features was used as input, ensuring that performance differences stem solely from model architecture.

#### 4.4.1. Robustness Evaluation with Random 5-Fold Cross-Validation

A preliminary comparison was conducted using stratified random 5-fold cross-validation, which assesses general performance when training and test samples are randomly distributed throughout the dataset. The quantitative results are summarized in Table 6.

The results in Table 6 show that MC-H-Geo achieved the highest overall accuracy (OA = 0.943) and Macro F1-score (0.945), outperforming all baseline methods by a substantial margin. Among the baselines, PointNet++ achieved the best performance (OA = 0.771), benefiting from its hierarchical feature aggregation directly from raw point clouds. SG-RFGeo performed moderately (OA = 0.742), benefiting from voxel-based 3D descriptors but limited by its fixed-scale representation of stratigraphic variability. XGBoost showed the lowest accuracy (OA = 0.617), highlighting that a single-stage classifier struggles to distinguish spectrally similar lithologies, even when trained on the full multi-scale feature set.

These results confirm that model architecture plays a decisive role in fine-grained geological classification. However, since random folds may include spatially adjacent points in both training and test sets, these metrics likely represent an optimistic upper bound of model performance. To obtain a more realistic assessment, a spatially disjoint cross-validation was subsequently performed (Section 4.4.2).

**Figure 14:**
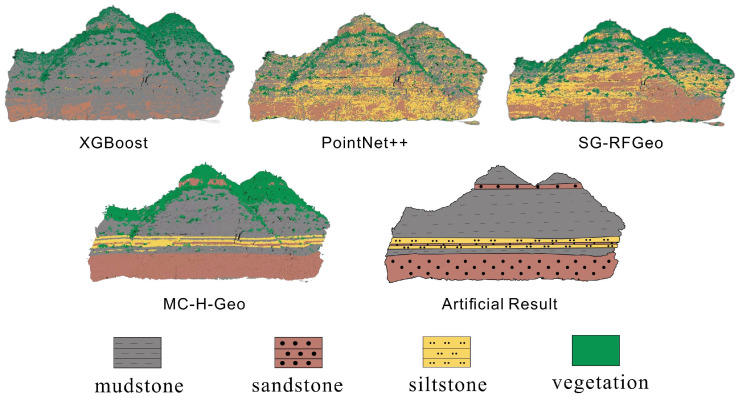
Comparative experimental results under random 5-fold cross-validation.

The visual results corresponding to this validation are shown in Figure 14. A qualitative analysis of the classification maps reveals the varying capabilities of each method. XGBoost produced coarse classifications with significant confusion between sandstone and mudstone intervals. SG-RFGeo delineated major units more clearly but exhibited fragmentation in fine strata and misclassification at vegetation-rock transitions. PointNet++ improved overall structure separation but retained considerable noise along lithological interfaces and produced fragmented vegetation boundaries. In stark contrast, MC-H-Geo generated the most consistent and continuous lithological mapping, with sharp boundaries that closely match the expert-labeled interpretations (“Artificial Result”).

While the results from random cross-validation provide a strong initial indication of MC-H-Geo’s superiority, this evaluation method can be susceptible to optimistic bias in geospatial datasets due to spatial autocorrelation. Therefore, a more stringent validation was performed.

#### 4.4.2. Robustness Evaluation with Spatial Cross-Validation

To obtain a more realistic and trustworthy assessment of the models’ generalization power, we implemented a spatial block cross-validation strategy. The entire labeled dataset was spatially divided into three contiguous, non-overlapping blocks of equal length along the outcrop’s primary horizontal axis. A 3-fold cross-validation was then performed, where in each fold, one entire block was held out as the test set, while the remaining two blocks were used for training. This ensures that the training and test data are always spatially disjoint. The comparative performance under this rigorous spatial validation scheme is presented in Table 7.

Compared with the random-split results in Table 6, all models exhibited decreased accuracy under the spatial scheme, reflecting the optimistic bias of conventional random validation and the challenge of cross-block generalization. Nevertheless, MC-H-Geo maintained a clear performance advantage (OA = 0.891, Macro F1 = 0.893) and superior class balance.

Across both validation schemes, the observed performance trends are geologically consistent and highlight the distinct capabilities and limitations of each method.

XGBoost exhibited the lowest accuracy because its single-stage learning structure failed to capture subtle intra-class variations. Although spectral amplitude and reflectance features are effective for vegetation–rock discrimination, they dominated the model, leading to strong confusion between spectrally similar lithologies such as mudstone and siltstone. This demonstrates that, for geological materials with overlapping spectral responses, model architecture is as critical as feature richness.

SG-RFGeo improved upon XGBoost by incorporating voxel-based 3D descriptors that better represent local spatial context. However, its reliance on fixed-scale, uniform voxels limited adaptability to heterogeneous stratigraphy. Sedimentary successions typically exhibit hierarchical structures—from centimeter-scale laminae to meter-scale bedding—and a single voxel size cannot capture these multiscale patterns simultaneously. As a result, SG-RFGeo often produced fragmented thin layers and boundary inconsistencies, particularly in interbedded mudstone–siltstone sequences.

PointNet++ achieved the best baseline performance due to its hierarchical aggregation of local geometric features directly from raw point clouds, enabling it to capture complex 3D morphologies. Nevertheless, without explicit multi-scale contextual features or stratigraphic constraints, it remained sensitive to local noise and failed to maintain lateral continuity across weathered or vegetation-affected zones.

In contrast, MC-H-Geo consistently delivered the most accurate, reliable, and geologically coherent results under both random and spatial validation. Its integrated design—combining multi-scale feature extraction, hierarchical task-specific classification, and stratigraphically informed post-processing—allows the model to bridge statistical learning and geological reasoning. This synergy enables MC-H-Geo to capture facies transitions across scales, suppress noise at lithological boundaries, and preserve the lateral and vertical continuity characteristic of real sedimentary architectures.

## 5. Discussion

The MC-H-Geo framework effectively addresses fine-grained lithological classification in complex outcrop point clouds by integrating multi-scale contextual feature extraction, hierarchical task-specific classification, and stratigraphically informed post-processing. On the Qianwangjiahe outcrop, it achieved an overall accuracy of 0.891 and a Macro F1-score of 0.893 under a rigorous spatial block cross-validation scheme. This performance significantly outperforms conventional machine learning, deep learning, and voxel-based baselines, while producing outputs that are highly consistent with expert interpretations of lithologic boundaries and stratigraphy (as shown in Section 4.4).

Multi-scale contextual features are crucial for resolving subtle lithological differences. As our feature importance analysis demonstrated, this is because different classification challenges rely on distinct physical properties captured at different scales. While the stark contrast in laser amplitude (driven by moisture and structure) effectively separates vegetation from rock, the discrimination among mudstone, siltstone, and fine sandstone relies on more subtle signatures of rock fabric. These signatures, rooted in differences in grain size, weathering resistance, and porosity, are best captured by a combination of geometric (e.g., point density, surface roughness) and textural descriptors. MC-H-Geo’s ability to extract and selectively utilize this rich, multi-scale information is fundamental to its success in resolving lithologies that exhibit overlapping spectral signatures due to their similar mineralogy.

The Hierarchical Gated Expert Classifier further leverages this enriched feature space by decomposing the task into coarse-to-fine stages. The coarse-level module robustly isolates vegetation from rock, allowing the fine-level expert module to focus on the more challenging task of discriminating lithologically similar classes. This expert module fuses the complementary strengths of Random Forest, XGBoost, and MLP classifiers via a logistic regression meta-learner. This structured approach is a key reason why MC-H-Geo maintains a high Macro F1-score even under the challenging spatial validation, in contrast to the significant performance drop seen in single-stage models like XGBoost (Table 7).

Stratigraphic post-processing provides the final, critical step by enforcing geological coherence. A *Z*-axis strata sweep maintains horizontal continuity, while neighborhood smoothing homogenizes thin interbeds, reducing patchy misclassification. This geology-aware refinement is essential for producing the continuous and coherent lithological boundaries seen in our final results (Figure 12, Scheme IV), a feature that purely data-driven methods like PointNet++ struggle to achieve consistently.

Compared with previous work on the Yueyawan outcrop [8], where coarse lithological contrasts (“0-to-1” problem) dominated, the Qianwangjiahe case represents a more challenging “1-to-1.1” scenario, requiring discrimination among fine-grained deltaic microfacies with subtle spectral, geometric, and textural differences. MC-H-Geo advances beyond SG-RFGeo by (i) introducing multi-scale contextual descriptors to capture microfacies variability, (ii) employing a fused hierarchical ensemble for task-specific feature allocation, and (iii) implementing stratigraphically informed post-processing without manual bias. These innovations deliver higher accuracy and geologically coherent results, particularly in subtle facies variations critical for reservoir characterization.

Regarding the amount of training data required for the classifier, our study utilized a substantial dataset of over 26,000 labeled points. The high and stable performance achieved through the rigorous spatial cross-validation process suggests that this data quantity was sufficient for the models to learn generalizable features without significant overfitting. While a formal learning curve analysis to determine the minimum required data was beyond the scope of this study, our results indicate that a dataset of this magnitude is adequate for building a robust classifier for this type of complex geological setting. Investigating model performance with smaller training subsets remains a valuable direction for future research, especially for scenarios where extensive data annotation is impractical.

**Figure 15:**
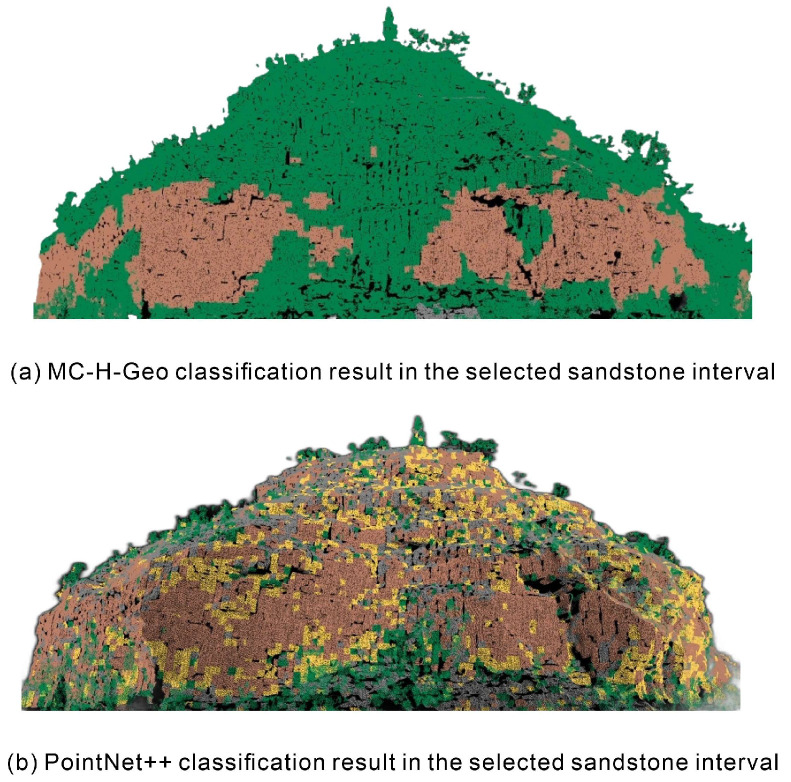
Representative comparison of classification performance in the upper sandstone interval between MC-H-Geo (**a**) and PointNet++ (**b**). Misclassification of sandstone as vegetation is highlighted in the MC-H-Geo result.

Despite these advances, residual errors persist, notably in upper sandstone units where laterally continuous strata are misclassified as vegetation (Figure 15). These errors arise from feature aliasing in weathered zones, where granular disaggregation, surface roughening, or anomalous mineralogy and moisture retention shift spectral–geometric signatures toward vegetation-like values. Interestingly, PointNet++ shows localized success in these ambiguous regions, suggesting that high-dimensional geometric cues exist that handcrafted features may not capture.

Future directions include: (i) advanced deep learning architectures, such as graph-based networks or transformer-based models, to learn subtle geometric patterns directly from point clouds, and (ii) multi-sensor data fusion. As astutely noted, color is a primary diagnostic feature for geological interpretation. Fusing our TLS-based framework with high-resolution RGB imagery, which was acquired during our survey, is the most direct path forward. This would not only allow for the derivation of vegetation indices (e.g., NDVI, NDRE) to virtually eliminate the residual rock-vegetation confusion but also enable the use of color and textural information to differentiate features like iron staining or weathering rinds, which are challenging to capture with laser intensity alone. Coupling this with hyperspectral imaging to provide detailed mineralogical information represents the next frontier in fully automated, geologically consistent digital outcrop mapping.

## 6. Conclusions

This study proposes MC-H-Geo, a comprehensive framework for fine-grained lithological classification of terrestrial laser scanning (TLS) outcrop point clouds. The workflow begins by voxelizing the raw point cloud into a 10 mm grid, from which geometric centroids are computed to generate anchor points that serve as stable analytical units. Based on these anchor points, a multi-scale contextual feature engine extracts spectral, geometric, and textural descriptors across multiple spatial resolutions, enabling the simultaneous capture of local variability and stratigraphic continuity. Lithological classification is then performed using a Gated Expert Classifier that applies task-adaptive, optimized feature subsets to hierarchical modules. Finally, a two-step geological post-processing strategy, consisting of stratigraphic correction along bedding-parallel axes and neighborhood-based smoothing, enhances both the spatial coherence and geological plausibility of the final classification maps.

From the experimental results obtained on the Qianwangjiahe outcrop, several conclusions can be drawn. First, the combination of multi-scale contextual features and hierarchical modeling substantially improves the recognition of lithologies with overlapping spectral–geometric signatures, reducing confusion between mudstone, sandstone, and siltstone. Second, the integration of stratigraphic priors through the two-step post-processing procedure enforces geological consistency, enabling accurate delineation of thin interbedded successions that baseline approaches fail to resolve. Third, detailed error analysis revealed that the residual misclassification of sandstone as vegetation stems from feature aliasing in weathered or biogenically altered zones, representing a fundamental physical limitation of TLS-only lithological classification. This finding not only reflects the current ceiling of feature-based methodologies but also delineates the intrinsic constraints of single-sensor outcrop analysis.

Looking ahead, two complementary directions appear most promising for overcoming these limitations. On the algorithmic side, advanced end-to-end deep learning architectures—potentially incorporating attention mechanisms or graph-based convolutions—could exploit subtle, high-dimensional spatial patterns that elude handcrafted descriptors. On the data side, multi-sensor fusion provides the most direct path forward. The integration of TLS with co-registered high-resolution RGB imagery would allow the derivation of vegetation indices to eliminate rock–vegetation confusion, while coupling with hyperspectral or multispectral imagery would provide diagnostic mineralogical information to resolve ambiguities within fine-grained successions.

In conclusion, the MC-H-Geo framework establishes a state-of-the-art methodology for automated, fine-grained lithological mapping of sedimentary outcrops. At the same time, its residual limitations highlight the necessity of combining advanced learning architectures with multi-sensor data fusion. Together, these developments represent the most promising route toward achieving fully automated, geologically consistent digital outcrop models across diverse depositional environments.

## Figures and Tables

**Table 1:** Technical specifications of the laser scanning instrument [8].

Model	Data Acquisition Rate (pts/s)	Field of View (°)	Range Accuracy (mm @ m)
RIEGL VZ400	500,000	360 × 100	±5 mm @ 50 m

**Table 2:** The number of samples of the collected dataset.

Class	Samples	Proportion (%)
Mudstone	8870	32.9
Sandstone	6010	22.6
Siltstone	6090	22.3
Vegetation	5940	22.2
**Total**	26,910	100

**Table 3:** Summary of the ultimate feature pool constructed for the MC-H-Geo framework.

Feature Category	Sub-Category	Number of Features	Representative Variables	Short Description
Spectral Statistics	Reflectance (raw)	13 × 2 = 26	ref_max, ref_std	Basic statistics of reflectance intensity.
	Normalized Reflectance	13 × 2 = 26	refnorm_mean, refnorm_cv	Intensity normalized for acquisition bias.
	Amplitude	13 × 2 = 26	amp_range, amp_cv	Return amplitude distribution descriptors.
Geometric Features	Roughness	5 × 2 = 10	roughness_sum, roughness_r0.1	Surface variation at multiple radii.
	Shape descriptors	3 × 2 = 6	linearity, planarity, sphericity	Eigenvalue-based 3D shape indices.
	Point density	1 × 2 = 2	point_density	Sampling density per anchor.
Textural Features (GLCM)	Reflectance-based	6 × 2 = 12	glcm_ref_contrast, glcm_ref_energy	Neighborhood texture of reflectance.
	Amplitude-based	6 × 2 = 12	glcm_amp_contrast, glcm_amp_ASM	Texture metrics from amplitude.
Frequency-domain (FFT)	Reflectance spectra	6 × 2 = 12	fft_ref_entropy, fft_ref_band_low	Frequency descriptors of reflectance.
	Amplitude spectra	6 × 2 = 12	fft_amp_peak_ratio, fft_amp_band_high	Frequency descriptors of amplitude.
Multi-scale Differential	Cross-scale differences/ratios	20	Δ(10–30 cm) of ref_std, planarity	Relative changes between fine and context scales.

Note: Only representative variables are shown in Table 3 to illustrate the main statistical descriptors within each sub-category. The complete enumeration of all variables (including reflectance, amplitude, normalized reflectance, geometric indices, textural measures, frequency-domain descriptors, and cross-scale differentials) is provided in Appendix A Table A1 and Table A2. Each feature type was calculated at two neighborhood scales (10 cm and 30 cm) centered on voxel-based anchor points (Section 2.2).Cross-scale differentials represent differences and ratios between fine- and context-scale descriptors, explicitly quantifying relative variability across scales.

**Table 4:** Optimized hyperparameter settings for the Gated Expert Classifier modules. The classifier consists of two task-specific modules: (i) a Random Forest–based Gating Module for robust vegetation–rock separation, and (ii) a stacking-based Expert Module integrating RF, XGBoost, and MLP, followed by a Logistic Regression meta-learner for fine-grained lithological discrimination. Reported values correspond to configurations selected through grid search with five-fold cross-validation.

Module	Model	Key Hyperparameter	Value	Purpose
Gating Module	Random Forest	*n_estimators*	300	Balance accuracy and computational efficiency
		*max_depth*	15	Prevents overfitting while capturing key feature
		*min_samples_leaf*	3	Ensure statistical robustness at each leaf node
		*class_weight*	balanced	imbalance between vegetation and rock samples
Expert Module	Random Forest	*n_estimators*	300	Improve sensitivity to subtle class differences
		*max_depth*	16	Enhance feature representation capability
	XGBoost	*n_estimators*	300	Provides gradient-boosted representation of non-linear patterns
		*learning_rate*	0.05	Controls step size shrinkage to improve generalization
		*reg_lambda (L2)*	6.0	Penalizes large weights, reducing model complexity
		*reg_alpha (L1)*	0.5	Encourages sparsity in feature usage
	MLP	*hidden_layer_sizes*	(128, 64)	Defines a two-layer neural architecture to capture non-linear relationships
		*alpha (L2)*	3 × 10^−4^	L2 penalty term for regularization
	Logistic Regression	*class_weight*	balanced	Mitigates imbalance among mudstone, sandstone, and siltstone

**Table 5:** Ablation experiment accuracy table.

	OA	Macro Precision	Macro Recall	Macro F1-Score
I	0.692	0.701	0.697	0.699
II	0.757	0.769	0.754	0.761
III	0.817	0.821	0.822	0.821
IV	0.943	0.945	0.944	0.945

**Table 6:** Performance comparison under standard random 5-fold cross-validation.

	OA	Macro Precision	Macro Recall	Macro F1-Score
XGBoost	0.617	0.623	0.621	0.624
PointNet++	0.771	0.781	0.774	0.779
SG-RFGeo	0.742	0.751	0.746	0.749
MC-H-Geo	0.943	0.945	0.944	0.945

**Table 7:** Performance comparison under 3-fold spatial block cross-validation.

	OA	Macro Precision	Macro Recall	Macro F1-Score
XGBoost	0.552	0.565	0.550	0.557
PointNet++	0.685	0.693	0.681	0.687
SG-RFGeo	0.661	0.670	0.659	0.664
MC-H-Geo	0.891	0.898	0.889	0.893

## Data Availability

The original contributions presented in the study are included in the article, further inquiries can be directed to the corresponding author. Codes and models that support this study are available at the GitHub link: https://github.com/WhLiuLang/MC-H-Geo. (accessed on 1 November 2025)”.

## Appendix A

### Appendix A.1

The complete enumeration of all variables (including reflectance, amplitude, normalized reflectance, geometric indices, textural measures, frequency-domain descriptors and cross-scale differentials) is provided in Table A1 and Table A2.

**Table A1:** Overview of extracted feature variables from TLS anchor points (Excerpt: Cross-scale Differentials).

Category	Feature Group	Variables	Description
Spectral statistics	Reflectance (10 cm/30 cm)	ref_max, ref_min, ref_range, ref_std, ref_mean, ref_median, ref_cv, ref_skew, ref_kurt, ref_q25, ref_q75, ref_iqr, ref_num_peaks	First-order descriptive statistics of reflectance values.
	Normalized reflectance (10 cm/30 cm)	refnorm_max, refnorm_min, refnorm_range, refnorm_std, refnorm_mean, refnorm_median, refnorm_cv, refnorm_skew, refnorm_kurt, refnorm_q25, refnorm_q75, refnorm_iqr, refnorm_num_peaks	Same metrics as above, normalized by distance, mitigating range attenuation.
Spectral statistics	Amplitude (10 cm/30 cm)	amp_max, amp_min, amp_range, amp_std, amp_mean, amp_median, amp_cv, amp_skew, amp_kurt, amp_q25, amp_q75, amp_iqr, amp_num_peaks	Pulse amplitude de-scriptors reflecting backscatter intensity.
Geometric indices	Roughness	roughness_sum, roughness_std, roughness_r0.05, roughness_r0.1, roughness_r0.2	Local roughness at mul-tiple neighborhood radii.
	Morphological shape	linearity, planarity, sphericity	Eigenvalue-based 3D shape descriptors.
	Point density	point_density	Sampling density, sensitive to porosity and occlusion.
Textural measures (GLCM)	GLCM from reflectance (10 cm/30 cm)	glcm_ref_contrast, glcm_ref_dissimilarity, glcm_ref_homogeneity, glcm_ref_ASM, glcm_ref_energy, glcm_ref_correlation	Second-order texture statistics from reflectance.
	GLCM from amplitude (10 cm/30 cm)	glcm_amp_contrast, glcm_amp_dissimilarity, glcm_amp_homogeneity, glcm_amp_ASM, glcm_amp_energy, glcm_amp_correlation	Same texture metrics from amplitude.
Frequency-domain descriptors (FFT)	Reflectance FFT (10 cm/30 cm)	fft_ref_energy_top1pct, fft_ref_peak_ratio, fft_ref_band_low, fft_ref_band_mid, fft_ref_band_high, fft_ref_spec_entropy	Frequency-based de-scriptors, energy dis-tri-bution across bands, and entropy.
	Amplitude FFT (10 cm/30 cm)	fft_amp_energy_top1pct, fft_amp_peak_ratio, fft_amp_band_low, fft_amp_band_mid, fft_amp_band_high, fft_amp_spec_entropy	Same descriptors for am-plitude.

Cross-scale differentials were computed as the difference or ratio between the corresponding 10 cm and 30 cm descriptors, enhancing sensitivity to stratigraphic variability.

**Table A2:** Representative cross-scale differential and ratio features derived from 10 cm and 30 cm neighborhoods.

Category	Feature Group	Representative Variables (Δ = 30–10 cm; R = 30 cm/10 cm)	Description
Cross-scale differentials (Δ = difference, Ratio = ratio)	Spectral (Ref, Refnorm, Amp)	Δref_mean, R_ref_std, Δamp_mean, R_amp_std	Captures changes in reflectance and amplitude statistics across scales.
	Geometric (Shape Indices)	Δroughness_std, Δlinearity, R_planarity, R_sphericity	Quantifies geometric variability between local and contextual neighborhoods.
	Point Density	Δpoint_density, R_point_density	Tracks scale-dependent sam-pling density differences.
	Textural (GLCM)	Δglcm_ref_contrast, R_glcm_amp_homogeneity	Measures contextual shifts in gray-level texture metrics.
Cross-scale differentials (Δ = difference, Ratio = ratio)	Frequency-domain (FFT)	Δfft_ref_spec_entropy, R_fft_amp_peak_ratio	Detects spectral energy re-distribution across scales.

Note: For each feature group, the dataset contains the complete set of differential (Δ) and ratio (R) metrics, including mean, std, median, quantiles, skewness, kurtosis, and peak-based descriptors. Only representative examples are listed here for compactness.

## Appendix B

### Appendix B.1

This appendix lists the 36 features selected by the Recursive Feature Elimination with Cross-Validation (RFECV) process for each of the two modules in the Gated Expert Classifier.

**Table A3:** Optimized Feature Subset for the Gating Module (Vegetation vs. Rock Classification).

Category	Feature Group	Variables	Count
Spectral statistics	Reflectance (10 cm)	ref_max, ref_min, ref_mean, ref_median, ref_q25, ref_q75	6
	Reflectance (30 cm)	ref_max, ref_min, ref_range, ref_mean, ref_median, ref_q25, ref_q75	7
	Amplitude (10 cm)	amp_max, amp_min, amp_mean, amp_median, amp_cv, amp_q25, amp_q75	7
	Amplitude (30 cm)	amp_max, amp_mean, amp_median, amp_q25, amp_q75	5
Geometric indices	Roughness (30 cm)	roughness_std, roughness_r0.05, roughness_r0.1,roughness_r0.2	4
	Morphological shape (10 cm)	sphericity	1
	Morphological shape (30 cm)	planarity, sphericity	2
	Point density (30 cm)	point_density	1
Textural measures (GLCM)	GLCM from amplitude (30 cm)	glcm_amp_correlation	1
Cross-scale differentials	Geometric (Roughness)	roughness_r0.2_diff	1
	Textural (GLCM)	glcm_ref_contrast_diff	1
Total			36

**Table A4:** Optimized Feature Subset for the Expert Module (Intra-Rock Classification).

Category	Feature Group	Variables	Count
Spectral statistics	Reflectance (10 cm)	ref_max	1
	Reflectance (30 cm)	ref_max, ref_range, ref_mean, ref_median, ref_cv, ref_q25, ref_q75	7
	Normalized reflectance (30 cm)	refnorm_std, refnorm_mean	2
	Amplitude (10 cm)	amp_max, amp_mean, amp_median,	3
Spectral statistics	Amplitude (30 cm)	amp_max, amp_min, amp_mean, amp_median, amp_q25, amp_q75	6
Geometric indices	Roughness (30 cm)	roughness_sum, roughness_std, roughness_r0.05, roughness_r0.1	4
	Point density (10 cm)	point_density	1
	Morphological shape (30 cm)	planarity, sphericity	2
	Point density (30 cm)	point_density	1
Textural measures (GLCM)	GLCM from amplitude (30 cm)	glcm_ref_contrast, glcm_ref_homogeneity, glcm_ref_ASM, glcm_amp_ASM	4
Cross-scale differentials/ratio	Normalized reflectance	refnorm_std_diff	1
	Geometric (Roughness)	roughness_sum_diff	1
	Textural (GLCM)	glcm_amp_correlation_diff, glcm_ref_homogeneity_ratio	2
	Frequency-domain (10 cm)	fft_ref_peak_ratio	1
Total			36
